# High-Throughput Precision Phenotyping of Left Ventricular Hypertrophy With Cardiovascular Deep Learning

**DOI:** 10.1001/jamacardio.2021.6059

**Published:** 2022-02-23

**Authors:** Grant Duffy, Paul P. Cheng, Neal Yuan, Bryan He, Alan C. Kwan, Matthew J. Shun-Shin, Kevin M. Alexander, Joseph Ebinger, Matthew P. Lungren, Florian Rader, David H. Liang, Ingela Schnittger, Euan A. Ashley, James Y. Zou, Jignesh Patel, Ronald Witteles, Susan Cheng, David Ouyang

**Affiliations:** 1 Department of Cardiology, Smidt Heart Institute, Cedars-Sinai Medical Center, Los Angeles, California; 2 Department of Medicine, Division of Cardiology, Stanford University, Stanford, California; 3 Department of Computer Science, Stanford University, Stanford, California; 4 National Heart and Lung Institute, Imperial College London, London, United Kingdom; 5 Department of Radiology, Stanford University, Stanford, California; 6 Department of Biomedical Data Science, Stanford University, Stanford, California; 7 Division of Artificial Intelligence in Medicine, Cedars-Sinai Medical Center, Los Angeles, California

## Abstract

**Importance:**

Early detection and characterization of increased left ventricular (LV) wall thickness can markedly impact patient care but is limited by under-recognition of hypertrophy, measurement error and variability, and difficulty differentiating causes of increased wall thickness, such as hypertrophy, cardiomyopathy, and cardiac amyloidosis.

**Objective:**

To assess the accuracy of a deep learning workflow in quantifying ventricular hypertrophy and predicting the cause of increased LV wall thickness.

**Design, Settings, and Participants:**

This cohort study included physician-curated cohorts from the Stanford Amyloid Center and Cedars-Sinai Medical Center (CSMC) Advanced Heart Disease Clinic for cardiac amyloidosis and the Stanford Center for Inherited Cardiovascular Disease and the CSMC Hypertrophic Cardiomyopathy Clinic for hypertrophic cardiomyopathy from January 1, 2008, to December 31, 2020. The deep learning algorithm was trained and tested on retrospectively obtained independent echocardiogram videos from Stanford Healthcare, CSMC, and the Unity Imaging Collaborative.

**Main Outcomes and Measures:**

The main outcome was the accuracy of the deep learning algorithm in measuring left ventricular dimensions and identifying patients with increased LV wall thickness diagnosed with hypertrophic cardiomyopathy and cardiac amyloidosis.

**Results:**

The study included 23 745 patients: 12 001 from Stanford Health Care (6509 [54.2%] female; mean [SD] age, 61.6 [17.4] years) and 1309 from CSMC (808 [61.7%] female; mean [SD] age, 62.8 [17.2] years) with parasternal long-axis videos and 8084 from Stanford Health Care (4201 [54.0%] female; mean [SD] age, 69.1 [16.8] years) and 2351 from CSMS (6509 [54.2%] female; mean [SD] age, 69.6 [14.7] years) with apical 4-chamber videos. The deep learning algorithm accurately measured intraventricular wall thickness (mean absolute error [MAE], 1.2 mm; 95% CI, 1.1-1.3 mm), LV diameter (MAE, 2.4 mm; 95% CI, 2.2-2.6 mm), and posterior wall thickness (MAE, 1.4 mm; 95% CI, 1.2-1.5 mm) and classified cardiac amyloidosis (area under the curve [AUC], 0.83) and hypertrophic cardiomyopathy (AUC, 0.98) separately from other causes of LV hypertrophy. In external data sets from independent domestic and international health care systems, the deep learning algorithm accurately quantified ventricular parameters (domestic: *R*^2^, 0.96; international: *R*^2^, 0.90). For the domestic data set, the MAE was 1.7 mm (95% CI, 1.6-1.8 mm) for intraventricular septum thickness, 3.8 mm (95% CI, 3.5-4.0 mm) for LV internal dimension, and 1.8 mm (95% CI, 1.7-2.0 mm) for LV posterior wall thickness. For the international data set, the MAE was 1.7 mm (95% CI, 1.5-2.0 mm) for intraventricular septum thickness, 2.9 mm (95% CI, 2.4-3.3 mm) for LV internal dimension, and 2.3 mm (95% CI, 1.9-2.7 mm) for LV posterior wall thickness. The deep learning algorithm accurately detected cardiac amyloidosis (AUC, 0.79) and hypertrophic cardiomyopathy (AUC, 0.89) in the domestic external validation site.

**Conclusions and Relevance:**

In this cohort study, the deep learning model accurately identified subtle changes in LV wall geometric measurements and the causes of hypertrophy. Unlike with human experts, the deep learning workflow is fully automated, allowing for reproducible, precise measurements, and may provide a foundation for precision diagnosis of cardiac hypertrophy.

## Key Points

**Question:**

Can deep learning be used to automate measurements of left ventricular dimensions and identify patients who could benefit from screening for underdiagnosed diseases?

**Findings:**

In this cohort study of 23 745 patients that used a deep learning algorithm to automatically measure left ventricular dimensions and to identify patients with increased wall thickness who may benefit from additional screening for hypertrophic cardiomyopathy and cardiac amyloidosis, the algorithm performed consistently across multiple cohorts while also delivering results in a shorter time than required for human assessment.

**Meaning:**

In this study, a deep learning workflow was able to automate wall thickness evaluation while facilitating identification of hypertrophic cardiomyopathy and cardiac amyloidosis.

## Teaser

This cohort study assesses the accuracy of a deep learning algorithm in measuring left ventricular dimensions and identifying patients with increased wall thickness who may benefit from additional screening for hypertrophic cardiomyopathy and cardiac amyloidosis.

## Introduction

Despite rapidly advancing developments in targeted therapeutics and genetic sequencing,^1,2^ persistent limits in the accuracy and throughput of clinical phenotyping has led to a widening gap between the potential and the actual benefits realized by precision medicine. This conundrum is exemplified by current approaches to assessing morphologic alterations of the heart.^3,4^ If reliably identified, misdiagnoses of certain cardiac diseases (eg, cardiac amyloidosis and hypertrophic cardiomyopathy [HCM]) could be avoided, and specific targeted therapies could be initiated efficiently. Systematic screening paradigms, including through imaging and automated medical record feature review, have shown the opportunity to identify patients with underdiagnosed diseases that are increasingly recognized as more prevalent than was previously thought.^5,6,7,8,9^ The ability to reliably distinguish between cardiac disease types with similar morphologic features but different causes would also enhance specificity for linking genetic risk variants and determining mechanisms.

The heart is a dynamic organ capable of remodeling and adapting to physiological stress and extracardiac perturbation. Both intrinsic cardiac disease and systemic insults can result in similar presentations of increased left ventricular (LV) wall thickness and LV hypertrophy (LVH), which are difficult to distinguish on routine imaging by human observation. Pressure overload from long-standing hypertension and aortic stenosis can cause cardiac remodeling to compensate for additional physiological work, and HCM and cardiac amyloidosis can similarly manifest with an increase in LV mass in the absence of physiological stress.^6,10^

In addition to the presence of LVH, the degree of ventricular thickness also has substantial prognostic value in many diseases.^10,11,12^ Ventricular wall thickness is used to risk-stratify patients for risk of sudden cardiac death and help determine which patients should undergo defibrillator implantation.^10^ Nevertheless, quantification of ventricular wall thickness remains subject to substantial intraprovider and interprovider variability across imaging modalities.^13,14^ Even with the high image resolution and signal-to-noise ratio of cardiac magnetic resonance imaging, there is marked test-retest variability owing to the laborious, manual nature of wall thickness measurement.^15,16^ Although abundant,^17,18^ low cost, and without ionizing radiation, echocardiography relies on expert interpretation, and its accuracy is dependent on careful application of measurement techinques.^19,20^

Recent work^21,22,23,24^ has shown that deep learning applied to medical imaging can identify clinical phenotypes beyond conventional image interpretation and with higher accuracy than interpretation by human experts. We hypothesized that echocardiography, the most common form of cardiovascular imaging on the basis of cardiology society guidelines for diagnosing hypertrophy including those of the European Society of Cardiology,^10^ when enhanced with artificial intelligence (AI) models, could provide additional value in understanding disease states by predicting both the presence and the potential cause of LVH in a screening population. To address current limitations in the assessment of ventricular hypertrophy and disease diagnosis, we developed an end-to-end deep learning approach for labeling the LV dimensions, quantifying ventricular wall thickness, and predicting the cause of LVH. We first conducted frame-level semantic segmentation of the left ventricular wall thickness from parasternal long-axis echocardiogram videos and then performed beat-to-beat evaluation of ventricular hypertrophy. After identifying LVH, we used a 3-dimensional convolutional neural network with residual connections to predict the cause of the LVH, including predictions for cardiac amyloidosis and aortic stenosis among a background of other hypertrophic diseases.

## Methods

### Data Curation

A standard full resting echocardiogram study consists of a series of 50 to 100 videos and still images visualizing the heart from different angles, locations, and image acquisition techniques (eg, 2-dimensional images, tissue Doppler images, and color Doppler images). In this cohort study, patients were identified in physician-curated cohorts from the Stanford Amyloid Center and Cedars-Sinai Medical Center (CSMC) Advanced Heart Disease Clinic for cardiac amyloidosis and the Stanford Center for Inherited Cardiovascular Disease and the CMSC Hypertrophic Cardiomyopathy Clinic for hypertrophic cardiomyopathy from January 1, 2008, to December 31, 2020. This research was approved by the Stanford University and CMSC institutional review boards. Written informed consent was obtained from patients for inclusion in the cohorts, but the need for consent for echocardiographic imaging analysis was exempted by the participating institutional review boards because of the use of deidentified images.

Relevant parasternal long-axis (PLAX) and apical 4-chamber 2-dimensional videos were extracted from each echocardiogram study (Table 1 and Table 2). Human clinician annotations of intraventricular septum (IVS), LV internal dimension (LVID), and LV posterior wall (LVPW) measurements were used as training labels to assess ventricular hypertrophy. Parasternal long-axis videos obtained from Stanford Health Care (SHC) were split and used as follows: 9600 for the training set, 1200 for the validation set, and 1200 for the test set. An additional 7767 SHC echocardiogram studies were from patients with defined disease characteristics, including cardiac amyloidosis, HCM, and severe aortic stenosis. From these studies, the apical 4-chamber videos were extracted and used as input data for the hypertrophic disease classification task. Videos were processed in a previously described automated preprocessing workflow in which identifying information and human labels had been removed.^21,25^

**Table 1.** Baseline Characteristics of Patients With Parasternal Long-Axis Videos of Echocardiograms From SHC and CSMC^a^

Characteristic	SHC				CSMC test set (n = 1309)
	Training set (n = 9601)	Validation set (n = 1200)	Test set (n = 1200)	Total (N = 12 001)	
Age, mean (SD), y	61.6 (17.4)	61.3 (17.8)	61.7 (17.5)	61.6 (17.4)	62.8 (17.2)
Sex					
Female	5268 (54.9)	618 (51.5)	623 (51.9)	6509 (54.2)	501 (38.3)
Male	4333 (45.1)	582 (48.5)	577 (48.1)	5492 (45.8)	808 (61.7)
Race and ethnicity					
American Indian	24 (0.2)	2 (0.2)	6 (0.5)	32 (0.3)	4 (0.3)
Asian	1377 (14.3)	176 (14.7)	180 (15.0)	1733 (14.4)	85 (6.5)
Black	373 (3.9)	53 (4.4)	50 (4.2)	476 (4.0)	239 (18.3)
Hispanic	1076 (11.2)	135 (11.2)	117 (9.8)	1328 (11.1)	178 (13.6)
Non-Hispanic White	4043 (42.1)	518 (43.2)	516 (43.0)	5077 (42.3)	697 (53.2)
Pacific Islander	140 (1.5)	14 (1.2)	23 (1.9)	177 (1.5)	4 (0.3)
Other^b^	742 (7.7)	98 (8.2)	96 (8.0)	936 (7.8)	83 (6.3)
Unknown	1826 (19.0)	204 (17.0)	212 (17.7)	2242 (18.7)	19 (1.5)
Atrial fibrillation	2067 (39.3)	281 (40.7)	258 (39.6)	2606 (39.5)	315 (24.1)
Congestive heart failure	3162 (60.1)	417 (60.3)	398 (61.1)	3977 (60.2)	451 (34.5)
Hypertension	3783 (71.9)	502 (72.6)	466 (71.6)	4751 (71.9)	543 (41.5)
Diabetes	1852 (35.2)	265 (38.4)	249 (38.2)	2366 (35.8)	248 (18.9)
Coronary artery disease	2517 (47.8)	343 (49.6)	316 (48.5)	3176 (48.1)	369 (28.2)
Chronic kidney disease	2066 (39.3)	265 (38.4)	252 (38.7)	2583 (39.1)	257 (19.6)
LVPWd thickness, mean (SD), cm	1.00 (0.21)	1.01 (0.21)	1.01 (0.21)	1.01 (0.21)	1.09 (0.25)
LVIDd, mean (SD), cm	4.70 (0.83)	4.69 (0.85)	4.71 (0.82)	4.70 (0.83)	4.70 (0.90)
LVIDs, mean (SD), cm	3.28 (0.90)	3.26 (0.90)	3.28 (0.88)	3.28 (0.90)	3.29 (1.05)
IVSd, mean (SD), cm	1.03 (0.24)	1.03 (0.24)	1.03 (0.25)	1.03 (0.24)	1.12 (0.29)
LVEF, mean (SD), %	55.71 (12.31)	55.62 (12.28)	56.08 (12.01)	55.74 (12.28)	55.92 (15.67)
LV mass, mean (SD), g	173.29 (68.71)	173.57 (69.97)	174.92 (68.84)	173.48 (68.84)	195.19 (83.16)

Abbreviations: CSMC, Cedars-Sinai Medical Center; IVSd, intraventricular septal thickness (diastole); LV, left ventricular; LVEF, left ventricular ejection fraction; LVIDd, left ventricular internal dimension (diastole); LVIDs, left ventricular internal dimension (systole); LVPWd, left ventricular posterior wall (diastole); SHC, Stanford Health Care.

^a^ Data are presented as number (percentage) of patients unless otherwise indicated.

^b^ Other racial and ethnic groups were not available because they were not included in the electronic health records.

**Table 2.** Baseline Characteristics of Patients With Apical 4-Chamber Videos of Echocardiograms From SHC and CSMC^a^

Characteristic	SHC				CSMC test set (n = 2351)
	Training set (n = 6461)	Validation set (n = 814)	Test set (n = 809)	Total (N = 8084)	
Cardiac amyloidosis	950 (14.7)	117 (14.4)	120 (14.8)	1187 (14.7)	358 (15.2)
Hypertrophic cardiomyopathy	2344 (36.3)	298 (36.6)	294 (36.3)	2936 (36.3)	146 (6.2)
Aortic stenosis	1061 (16.4)	133 (16.3)	132 (16.3)	1326 (16.4)	468 (19.9)
Other LVH	2106 (32.6)	266 (32.7)	263 (32.5)	2635 (32.6)	1379 (58.7)
Age, mean (SD), y	69.0 (16.9)	70.0 (16.6)	69.4 (16.0)	69.1 (16.8)	69.6 (14.7)
Sex					
Female	3352 (51.9)	414 (50.9)	435 (53.8)	4201 (52.0)	731 (31.1)
Male	3109 (48.1)	400 (49.1)	374 (46.2)	3883 (48.0)	1620 (68.9)
Race and ethnicity					
American Indian	11 (0.2)	1 (0.1)	1 (0.1)	13 (0.2)	4 (0.2)
Asian	586 (9.1)	77 (9.5)	53 (6.6)	716 (8.9)	105 (4.5)
Black	252 (3.9)	24 (2.9)	37 (4.6)	313 (3.9)	353 (15.0)
Hispanic	551 (8.5)	59 (7.2)	56 (6.9)	666 (8.2)	266 (11.3)
Non-Hispanic White	2895 (44.8)	387 (47.5)	346 (42.8)	3628 (44.9)	1468 (62.4)
Pacific Islander	58 (0.9)	7 (0.9)	2 (0.2)	67 (0.8)	3 (0.1)
Other^b^	666 (10.3)	86 (10.6)	96 (11.9)	848 (10.5)	146 (6.2)
Unknown	1442 (22.3)	173 (21.3)	218 (26.9)	1833 (22.7)	6 (0.3)
Atrial fibrillation	2209 (49.5)	293 (51.3)	262 (50.0)	2764 (49.8)	630 (26.8)
Congestive heart failure	3632 (81.5)	467 (81.8)	422 (80.5)	4521 (81.4)	876 (37.3)
Hypertension	3657 (82.0)	473 (82.8)	423 (80.7)	4553 (82.0)	1290 (54.9)
Diabetes	1462 (32.8)	178 (31.2)	180 (34.4)	1820 (32.8)	478 (20.3)
Coronary artery disease	2858 (64.1)	359 (62.9)	324 (61.8)	3541 (63.8)	840 (35.7)
Chronic kidney disease	2238 (50.2)	297 (52.0)	292 (55.7)	2827 (50.9)	513 (21.8)
LVPWd thickness, mean (SD), cm	1.18 (0.25)	1.19 (0.24)	1.17 (0.24)	1.18 (0.25)	1.44 (0.26)
LVIDd, mean (SD), cm	4.58 (0.75)	4.61 (0.75)	4.58 (0.72)	4.58 (0.75)	4.52 (0.86)
LVIDs, mean (SD), cm	3.15 (0.78)	3.19 (0.81)	3.17 (0.77)	3.15 (0.78)	3.07 (0.81)
IVSd, mean (SD), cm	1.27 (0.31)	1.28 (0.31)	1.26 (0.32)	1.27 (0.31)	1.52 (0.37)
LVEF, mean (SD), %	56.92 (11.70)	56.50 (11.61)	56.73 (12.03)	56.86 (11.72)	59.06 (13.68)
LV mass, mean (SD), g	215.73 (80.61)	219.52 (76.97)	213.66 (78.67)	215.91 (80.06)	278.64 (96.53)

Abbreviations: CSMC, Cedars-Sinai Medical Center; IVSd, intraventricular septal thickness (diastole); LV, left ventricular; LVEF, left ventricular ejection fraction; LVIDd, left ventricular internal dimension (diastole); LVIDs, left ventricular internal dimension (systole); LVPWd, left ventricular posterior wall (diastole); SHC, Stanford Health Care.

^a^ Data are presented as number (percentage) of patients unless otherwise indicated.

^b^ Other racial and ethnic groups were not available because they were not included in the electronic health records.

### Domestic and International External Health Care System Test Data Sets

Transthoracic echocardiogram studies from CSMC and the Unity Imaging Collaborative^16^ were used to evaluate the deep learning algorithm’s performance in identifying key points in PLAX videos and measuring ventricular dimensions. Previously described methods were used to identify PLAX and apical 4-chamber–view videos and to convert Digital Imaging and Communications in Medicine files to AVI files.^22^ We extracted a total of 3660 videos from CSMC as a domestic held-out test data set. Labeled images from the Unity Imaging Collaborative were used as an additional held-out international test data set not seen during model training. These echocardiogram videos were obtained from the British echocardiography laboratories and were retrospectively annotated by echocardiography-certified cardiologists.

### Deep Learning Algorithm Development and Training

Model design and training were done in Python, version 3.8.5 (Python Software Foundation) using the PyTorch deep learning library. A modified DeepLabv3^26^ architecture trained on parasternal long-axis images to minimize a weighted mean square error loss was used to identify key points used for measuring ventricular dimensions. Three-dimensional implementations of a segmentation model took substantially more computational resources without marked improvement in performance. An Adam optimizer with a learning rate of 0.001 was used, and the model was trained for 50 epochs, with early stopping based on the validation loss. We evaluated different video lengths, resolutions, and temporal resolutions as hyperparameters to optimize model performance. Computational cost was evaluated using 1 NVIDIA GeForce GTX 3090.

For video-based disease classification, an 18-layer ResNet3D^27^ architecture was used to classify videos. Given the potential for patients who had diagnoses of both aortic stenosis and cardiac amyloidosis with multiple causes of LVH,^28^ parallel binary classification deep learning models were trained to predict probability of cardiac amyloidosis, HCM, and aortic stenosis secondary to uncontrolled hypertension and in the context of end-stage kidney disease independently. Distinct from previous literature,^7,8^ for each classification task, the negative controls were images from patients with other causes of LVH to mimic the clinical workflow. For example, during amyloid classification, the negative training examples included videos from patients with diagnosed HCM, aortic stenosis, hypertension, and end-stage kidney disease as other causes of LVH. This model was trained to minimize binary cross-entropy loss using an Adam optimizer with a learning rate of 0.01. The model was trained for 100 epochs with a batch size of 14, with early stopping based on area under the curve (AUC) on the validation set (eMethods in the Supplement).

### Comparison With Variation in Human Measurement

Using the reporting database of the Stanford Echocardiography Laboratory, we identified paired studies of the same patient for which the reviewing cardiologist determined there was no substantial change from the current study to the previous study by a structured reporting element. Of these studies with clinical stability, we analyzed the subset of 23 874 studies for which LVID, IVS, and LVPW at diastole were measured for both the current and the subsequent studies. The variance in measurement between the previous and subsequent studies was used as a surrogate of clinician variation and was compared with the variation of the deep learning algorithm. In the CSMC data set, we identified 99 random studies; blinded relabeling was performed by 2 level 3 echocardiography-certified cardiologists, and their performance was compared with the performance of the deep learning algorithm on the consensus label.

### Statistical Analysis

The 95% CIs were computed using 10 000 bootstrapped samples and by obtaining 95th percentile ranges for each prediction. The performance of the semantic segmentation task was evaluated by comparing the length of LVID, LVPW, and IVS with human labels in the hold-out test data set. The centroid of each predicted key point was used to calculate measurements with Python software, version 3.8.5.

## Results

**Figure 1.**
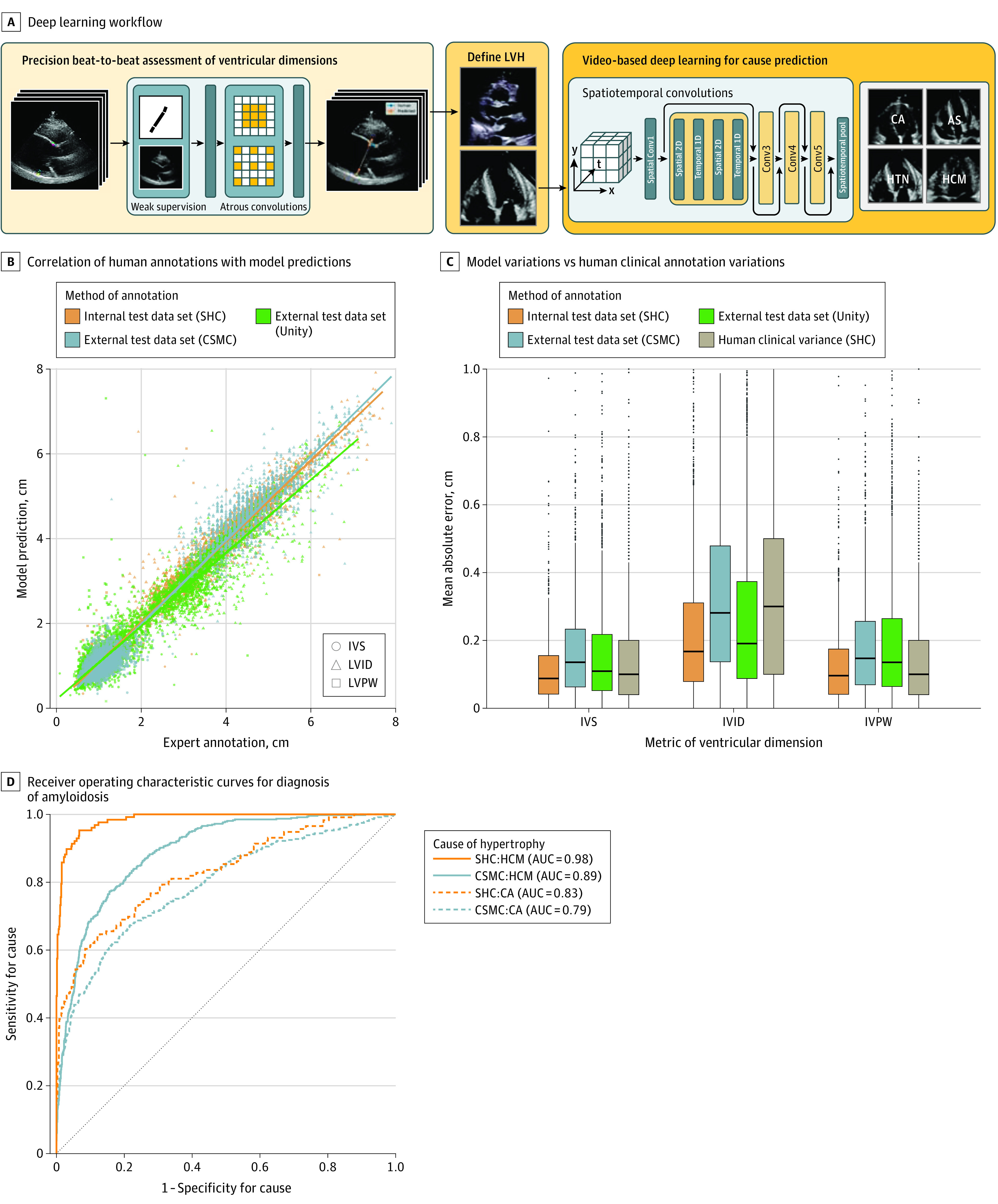
Deep Learning Workflow Combining Evaluation of Ventricular Dimensions and Suspicion for Underdiagnosed Diseases A, The deep learning algorithm used parasternal long-axis echocardiogram video as input to derive key points and estimate ventricular dimensions. After identifying patients with left ventricular hypertrophy (LVH), the deep learning workflow used a video-based architecture to distinguish common causes of LVH. B, Correlation of human annotations vs model predictions for ventricular dimensions in data sets from Stanford Health Care (SHC; n = 1200), Cedars-Sinai Medical Center (CSMC; n = 1309), and Unity Imaging Collaborative (n = 1791). C, Model variation on the 3 data sets vs human clinical annotation variation. Middle lines represent means; upper and lower bounds of the boxes, 25th and 75th percentiles; and points, values greater than 1.5 times the IQR. D, Receiver operating characteristic curves for diagnosis of amyloidosis in the SHC validation (n = 813) and test (n = 812) sets. AS indicates aortic stenosis; AUC, area under the curve; CA, cardiac amyloid; HCM, hypertrophic cardiomyopathy; HTN, hypertension; IVS, intraventricular septum; LVID, LV internal dimension; LVPW, LV posterior wall.

**Figure 2.**
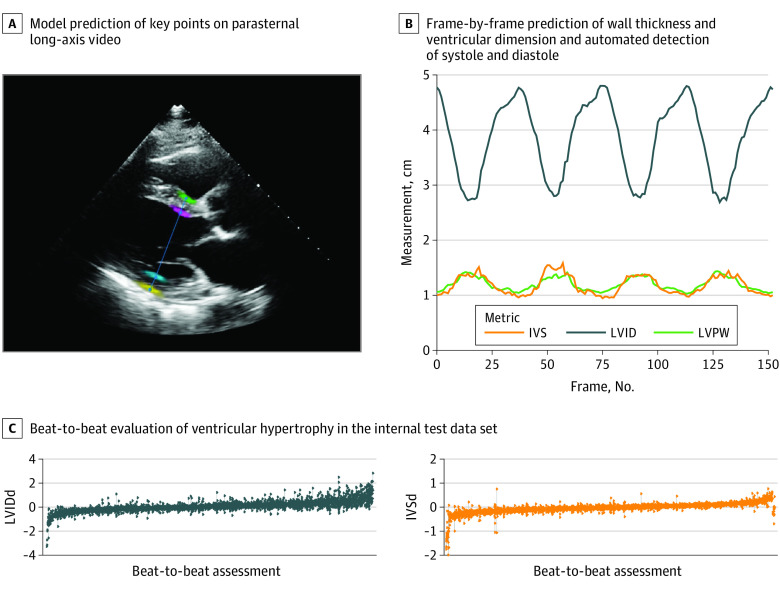
Beat-to-Beat Evaluation of Ventricular Dimensions A, Model prediction of key points on an individual frame of parasternal long-axis video. B, Frame-by-frame prediction of wall thickness and ventricular dimension and automated detection of systole and diastole allowing for beat-to-beat prediction of ventricular hypertrophy. C, Waterfall plot of individual video variation in beat-to-beat evaluation of ventricular hypertrophy (n = 2320) in the internal test data set. Each video is represented by multiple points along a line representing the measurement of each beat and a line signifying the range of predictions. IVS indicates intraventricular septum; IVSd, intraventricular septum (diastole); LVID, left ventricular internal dimension; LVIDd, left ventricular internal dimension (diastole); LVPW, left ventricular posterior wall.

The study included 23 745 patients: 12 001 from SHC (6509 [54.2%] female; mean [SD] age, 61.6 [17.4] years) and 1309 from CSMC (808 [61.7%] female; mean [SD] age, 62.8 [17.2] years) with parasternal long-axis videos and 8084 from SHC (4201 [54.0%] female; mean [SD] age, 69.1 [16.8] years) and 2351 from CSMS (6509 [54.2%] female; mean [SD] age, 69.59 [14.7] years) with apical 4-chamber videos. The deep learning workflow for screening of HCM and cardiac amyloidosis had 2 components (Figure 1). First, we designed a deep learning model with atrous convolutions for semantic segmentation of PLAX echocardiogram videos and identification of the IVS, LV internal dimension, and LV posterior wall. With atrous convolutions to capture longer-range features, full-resolution PLAX frames were used as input images for higher-resolution assessment of LVH. Given the tedious nature of annotation, in the standard clinical workflow, only 1 or 2 frames of a video are often labeled, but each video records multiple heartbeats that can be used for clinical measurements (Figure 2). Therefore, we generalized a neural network trained on these sparse annotations into measurement predictions for every frame of the entire video to allow for beat-to-beat estimation of ventricular wall thickness and dimensions. Representative example parasternal long axis videos with model annotations are shown in the Video.

**Video.** Deep Learning Prediction of Left Ventricular Dimensions and Screening for Cardiac Amyloidosis and Hypertrophic Cardiomyopathy Representative parasternal long-axis videos with frame-by-frame deep learning annotations. Colored regions indicate probability distribution of each key point predicted by the model. Blue lines are maximum likelihood predictions of ventricular dimensions.

**Figure 3.**
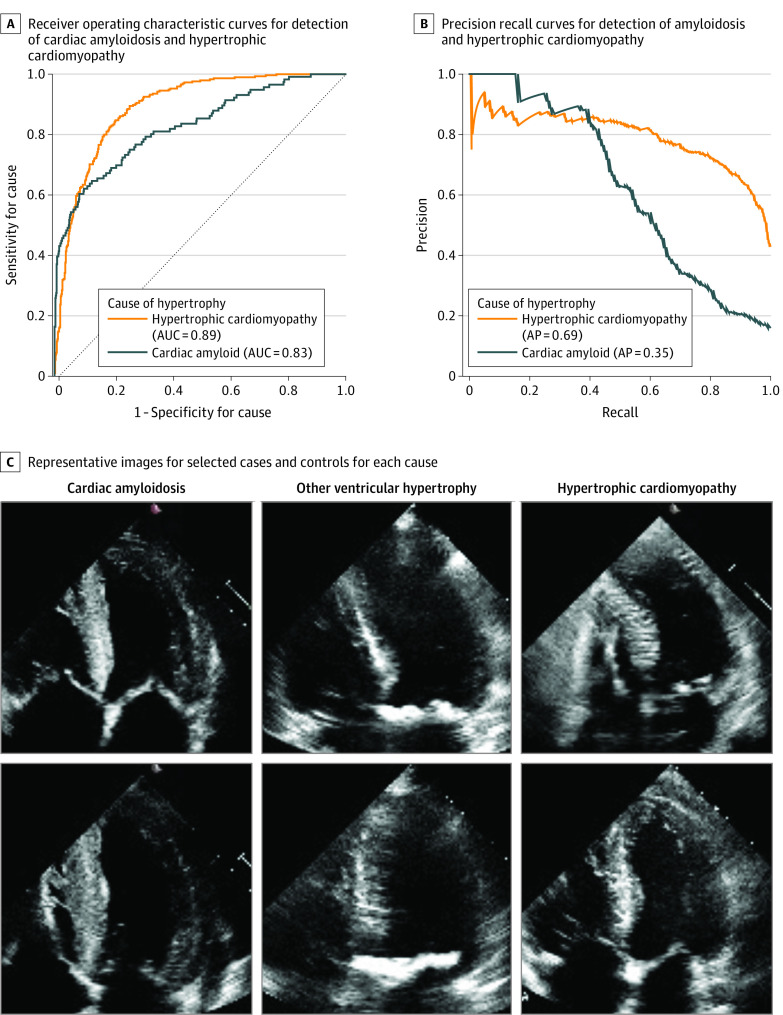
Performance of Disease Cause Classification in the Independent External Validation Cohort A, Receiver operating characteristic curves for detection of cardiac amyloidosis and hypertrophic cardiomyopathy in the Cedars-Sinai Medical Center independent external test set (n = 2351). B, Precision-recall curves for detection of amyloidosis and hypertrophic cardiomyopathy. C, Representative images for selected cases and controls for each cause. AP indicates average precision; AUC, area under the curve.

After detection of LVH, identifying the specific cause (eg, infiltrative disease, inherited cardiomyopathies, or chronic elevated afterload) can help guide therapy. We trained a video-based convolutional neural network model with spatiotemporal convolutions to predict the cause of LVH (Figure 3). Integrating spatial and temporal information, the model expanded on previous work^21^ with video-based model interpretation of echocardiograms and classified videos based on probability of hypertension, aortic stenosis, HCM, or cardiac amyloidosis as causes of ventricular hypertrophy. In addition, we performed a video-based model architecture and hyperparameter search to identify the optimal base architecture for the deep learning algorithm (eFigure 1 in the Supplement). The deep learning algorithm was trained on a data set of 17 802 echocardiogram videos from SHC and then evaluated on held-out test cohorts from SHC, CSMC, and Unity Imaging Collaborative.

### Evaluation of Hypertrophy Detection

From the held-out test data set from SHC not seen during model training (n = 1200), the deep learning algorithm predicted ventricular dimensions with an *R*^2^ of 0.97 compared with annotations by human experts (eFigure 2 in the Supplement). The deep learning algorithm had a mean absolute error (MAE) of 1.2 mm (95% CI, 1.1-1.3 mm) for IVS thickness, 2.4 mm (95% CI, 2.2-2.6 mm) for LVID, and 1.4 mm (95% CI, 1.2-1.5 mm) for LVPW thickness. This compared favorably with clinical inter-provider variation, which had an MAE of 1.3 mm (95% CI, 1.3-1.3 mm) for IVS thickness, 3.7 mm (95% CI, 3.6-3.7 mm) for LVID, and 1.3 mm (95% CI, 1.3-1.3 mm) for LVPW thickness. The deep learning algorithm also performed well compared with the prospective consensus annotation of 2 level-3 echocardiography-certified cardiologists in 99 random studies from CSMC (eFigure 3 in the Supplement). To assess the reliability of the model across health care systems internationally, the deep learning algorithm was also tested without any tuning on an external test data set of 1791 videos from Unity Imaging Collaborative and 3660 videos from CSMC. On the Unity Imaging Collaborative external test data set, the deep learning algorithm showed a robust prediction accuracy, with an overall *R*^2^ of 0.90 and MAEs of 2.2 mm (95% CI, 1.7-2.6 mm) for IVS thickness, 4.5 mm (95% CI, 3.7-5.3 mm) for LVID, and 2.4 mm (95% CI, 2.0-2.7 mm) for LVPW thickness. When fine-tuned using the training split of the Unity Imaging Collaborative data set, the deep learning algorithm showed an improved performance with an overall *R*^2^ of 0.92 and MAEs of 1.7 mm (95% CI, 1.5-2.0 mm) for IVS thickness, 2.9 mm (95% CI, 2.4-3.3 mm) for LVID, and 2.3 mm (95% CI, 1.9-2.7 mm) for LVPW thickness on the Unity Imaging Collaborative validation data split, indicating data shift and potential variations in practice across institutions and continents (eTable 1 in the Supplement).

A rapid, high-throughput automated approach allowed for measurement of every individual frame, which would be tedious for manual tracing (Figure 2). Differences in filling time and irregularity in the heart rate can cause variation in measurement, but beat-to-beat model assessment can provide higher-fidelity overall assessments. Although the SHC and Unity Imaging Collaborative data sets were directly compared on annotated individual frames, we evaluated the deep learning algorithm’s beat-to-beat evaluation on the CSMC data set compared with study-level annotations of ventricular dimensions. In this data set, human measurements were not associated with specific frames of the echocardiogram video, and beat-to-beat analysis was used to predict diastole and mean measurements from each heartbeat throughout the entire video. On the CSMC external test data set, the deep learning algorithm showed a robust prediction accuracy with an overall *R*^2^ of 0.96 and MAEs of 1.7 mm (95% CI, 1.6-1.8 mm) for IVS thickness, 3.8 mm (95% CI, 3.5-4.0 mm) for LVID, and 1.8 mm (95% CI, 1.7-2.0 mm) for LVPW thickness with beat-to-beat evaluation.

### Prediction of Cause of LVH

The cause derivation, validation, and test cohorts from SHC had 6215, 787, and 765 videos, respectively. In the held-out test cohort, the deep learning algorithm distinguished cardiac amyloidosis with an AUC of 0.83, HCM with an AUC of 0.98, and aortic stenosis with an AUC of 0.89 from other causes of LVH. On the held-out test cohort, the area under the precision-recall curve of the deep learning algorithm was 0.77 for cardiac amyloidosis, 0.95 for HCM, and 0.79 for aortic stenosis. The proposed ensemble of binary classification video-based deep learning classifiers in the deep learning algorithm was similar in performance to a multilabel, multiclass deep learning model for disease detection but had the flexibility of being able to identify patients who had diagnoses of both aortic stenosis and cardiac amyloidosis. In an external test data set of 2351 apical 4-chamber–view videos from CSMC with 358 videos of cardiac amyloidosis, 146 videos of aortic stenosis, 468 videos of HCM, and 1379 videos of other causes of LVH, the deep learning algorithm had an AUC of 0.79 for predicting cardiac amyloidosis and an AUC of 0.89 for predicting HCM. For the CSMC cohort, the area under the precision-recall curve of the deep learning algorithm was 0.54 for cardiac amyloidosis, 0.69 for HCM, and 0.08 for aortic stenosis. The model performance was consistent across body mass index and image quality (eTable 2 in the Supplement).

### Phenotypic Mimics and Disease-Specific Training Pipeline

To highlight the benefit of training a model with negative controls derived from other causes of LVH instead of normal controls, we performed a series of experiments to see how a model that was trained without seeing other phenotypic mimics would perform when encountering phenotypic mimics. A confusion matrix was generated in the 2 experimental settings (eTable 3 in the Supplement) in which a higher AUC outside the diagonal showed the model confusion and a lower AUC suggested improved discrimination between phenotypic mimics. In this experiment, although the model produced a higher AUC (0.96 for cardiac amyloidosis, 0.98 for aortic stenosis, and 0.97 for HCM), there was substantial confusion when other causes were introduced, suggesting that a model trained only on age- and sex-matched controls would primarily identify LVH.

## Discussion

The AI-guided workflow used in this cohort study was a deep learning system that automatically quantified LV wall thickness on echocardiography while also predicting the cause of LVH as either HCM or cardiac amyloidosis. The deep learning algorithm performed measurements of ventricular thickness and diameter well within the variance of human clinical test-retest assessment while aiding the detection of subtle ventricular phenotypes that tend to be challenging for human readers. This integration of LV measurement and prediction of cause offers an automated workflow for disease screening from echocardiography, the most frequently used form of cardiac imaging. As such, echocardiography-based screening can provide a high index of suspicion that can facilitate more efficient clinical evaluation, diagnosis, and care. Assimilation of automated diagnostic algorithms with widely available clinical imaging can reduce physician burden while streamlining opportunities for more targeted cardiovascular care.

Studies^28,29,30^ have suggested that diseases such as cardiac amyloidosis are underdiagnosed rather than rare. Given the large heterogeneous population of patients with heart failure with preserved ejection fraction,^31^ methods that might appropriately and efficiently increase suspicion for under-recognized causes, such as subtypes of amyloidosis with newly available targeted therapies, may help address a persistent unmet need. Accordingly, an opportunity exists in the application of efficient AI algorithms to increase recognition of historically underdiagnosed diseases in stored images in databases of large echocardiography laboratories. Notwithstanding that all patient data should be interpreted in the clinical context, our findings suggest that automated image analysis workflows could be feasibly implemented to rapidly identify patients who could benefit from follow-up screening in large populations. As such, more prospective work is needed to evaluate the potential of such algorithms to expedite appropriate clinical evaluation, targeted testing, and confirmation before eventual diagnosis and initiation of disease-modifying therapy.

The results of the present study represent a step toward the automated assessment of cardiac structures in echocardiogram videos through deep learning. Although individual linear measurements take only seconds to measure, there is inherent variation in frame and video selection that sets a floor to the precision of manual measurements derived from echocardiography. Future work should augment echocardiographic labels with annotations and information from cardiac magnetic resonance imaging and other imaging modalities to have more precision automation. By using an automated method, potentially more precise measurements can be obtained in busy clinical and research settings. Combined with previous work^8^ assessing cardiac function, the present study showed that deep learning models on echocardiogram images can automate an increasingly larger proportion of tasks for assessing cardiac function and structure to provide more holistic evaluation of cardiovascular disease. With improved precision to detect ventricular remodeling and cardiac dysfunction, AI systems offer the potential for earlier detection and treatment of subclinical cardiovascular disease, including less common or underdiagnosed conditions.

### Strengths and Limitations

This study has several strengths. A key challenge in the use of AI in health care has been the lack of benchmark data sets for direct comparison of models and engineering workflows across institutions. Data set inclusion criteria, differences in annotations and disease definitions, and protocols of how to annotate images are sources of data set shift that limit the direct comparison of model performance.^32,33^ With fine-tuning on site-specific data, our model compares favorably with prior state-of-the-art approaches to assessing ventricular wall thickness and hypertrophy on open benchmarks.^16^ Expanding on previous work,^21^ we collaborated with stakeholders across Stanford Medicine to release our data set of 12 000 deidentified PLAX echocardiogram videos as a resource for the medical machine learning community for future comparison and validation of deep learning models. This expands the prior data set of 10 030 apical 4-chamber videos^21^ to a total of 22 030 echocardiogram videos made publicly available, which to our knowledge, is the largest data set release of labeled medical videos with matched clinician annotations. We hope this data set will facilitate new echocardiogram- and medical video–based machine learning approaches. We also released the full code for our algorithm and data-processing workflow.

This study also has limitations. First, because the training images for this study were obtained from curated cohorts of patients from tertiary care specialty clinics, biases in patient selection for these clinics can be present in deployment of such algorithms. For example, although hereditary cardiac amyloidosis is known to disproportionately affect Black individuals in the US, they are underrepresented in study cohorts, and care must be taken to extrapolate performance of deep learning algorithms in populations with different demographic characteristics.^34,35^ Second, our model was trained on videos obtained by expert sonographers at an academic medical center. With expansion in the use of point-of-care ultrasonography for evaluation of cardiac function by noncardiologists, further work is needed to understand model performance with input videos of more variable quality and acquisition expertise as well as in comparison with other imaging modalities. Although our analyses across health systems suggest that our deep learning algorithm is robust to variation in practice patterns across continents, prospective deployment and testing of AI systems in diverse clinical environments remain to be done. A key limitation of research in this field has been a dearth of prospective trials and evaluation of model performance during clinical deployments.^34^ As such, further work and prospective validation are needed to more fully understand the effect of AI-guided screening workflows on clinical care.

### Conclusions

In this cohort study, using measurements across multiple heartbeats and validated against 3 international cohorts, the deep learning model more accurately identified subtle changes in LV wall geometric measurements than did human assessment and accurately identified the cause of LVH. A rapid, fully automated workflow, the deep learning algorithm allows for reproducible, precise measurements and may provide the foundation for precision diagnosis of cardiac hypertrophy.

## Notes

Supplement. **eMethods**. **eFigure 1.** Hyperparameter Search of Video-based Classification Model **eFigure 2.** Comparison of Model Performance With Human Variation **eFigure 3.** Comparison of Model Performance With Prospective Consensus Annotation of Two Level III Echocardiography Certified Cardiologists **eTable 1.** Performance on Unity Imaging Collaborative External Test Data set With and Without fine-tuning **eTable 2.** Performance Across Body Mass Index **eTable 3.** Minimizing Confusion of Alternative Etiologies of Hypertrophy When Trained on Age and Sex Matched Control Cases Vs Other Hypertrophic Control
