# Supplementary material for: High-Throughput Precision Phenotyping of Left Ventricular Hypertrophy With Cardiovascular Deep Learning
Source: JAMA Cardiol. 2022 Feb 23;7(4):386–95. doi: 10.1001/jamacardio.2021.6059 (PMC9008505; doi:10.1001/jamacardio.2021.6059)
Supplement: Supplement. — eMethods. eFigure 1. Hyperparameter Search of Video-based Classification Model eFigure 2. Comparison of Model Performance With Human Variation eFigure 3. Comparison of Model Performance With Prospective Consensus Annotation of Two Level III Echocardiography Certified Cardiologists eTable 1. Performance on Unity Imaging Collaborative External Test Data set With and Without fine-tuning eTable 2. Performance Across Body Mass Index eTable 3. Minimizing Confusion of Alternative Etiologies of Hypertrophy When Trained on Age and Sex Matched Control Cases Vs Other Hypertrophic Control [file jamacardiol-e216059-s001.pdf]

Supplementary Online Content

Duffy G, Cheng PP, Yuan N, et al. High-throughput precision phenotyping of left ventricular hypertrophy with cardiovascular deep learning. *JAMA Cardio.* Published online February 23, 2022. doi:10.1001/jamacardio.2021.6059

eMethods.
eFigure 1. Hyperparameter Search of Video-based Classification Model
eFigure 2. Comparison of Model Performance With Human Variation
eFigure 3. Comparison of Model Performance With Prospective Consensus Annotation of Two Level III Echocardiography Certified Cardiologists
eTable 1. Performance on Unity Imaging Collaborative External Test Dataset With and Without fine-tuning
eTable 2. Performance Across Body Mass Index
eTable 3. Minimizing Confusion of Alternative Etiologies of Hypertrophy When Trained on Age and Sex Matched Control Cases Vs Other Hypertrophic Control

This supplementary material has been provided by the authors to give readers additional information about their work.

© 2022 Duffy G et al. *JAMA Cardiology*.

eMethods.

Ventricular Dimension Calculation

Sparse human clinician annotations were used for training of semantic segmentation task of identifying key points along ventricular septum and posterior wall to calculate IVS, LVID, and LVPW. For each video, only one frame was labeled at diastole and systole. Each key point (four in total) was mapped to four output channels of a DeepLabV3 architecture with a gaussian sampling to simulate human variation. The gaussian sampling was performed by the data loader during training and standard deviation of the gaussian sampling was empirically sampled as a model hyperparameter. The labels were four channel images with all zeros except ones for pixels nearest to the ground truth measurement point in each channel.

The loss function used to train the PLAX model is a modified mean squared error loss augmented with the L2 loss for point location error and measurement error. Due to the sparse nature of the labels (roughly only 4 pixels in 480x640 total pixels per frame), the modified mean squared error loss allows us to independently penalize false positive loss and false negative loss by a hyperparameter that can be chosen based on class imbalance.

$$l_{wmse} = \frac{1}{n}\sum_{i=0}^{n}[\alpha(1 - y_i)(y_i - \hat{y_i})^2 + (1 - \alpha)y_i(y_i - \hat{y_i})^2]$$

An alpha value of 0.001 and an augmented L2 loss weight of 0.001 were chosen through experimentation and hyperparameter sweeps. This allows the model to predict more pixels with high confidence improving stability, training time and interpretability.

The centroid of each output channel is used as the endpoints for each measurement. In beat-to-beat evaluation, heuristics were used to exclude low quality frames of the video from overall calculation. At inference time, low confidence pixels (with a score less than 0.3) were ignored in calculating the centroid. If the model predicts measurements that are inconsistent in angle (greater than 30 degrees different), the frame is considered low quality and the prediction is ignored during video-wide averaging.

© 2022 Duffy G et al. *JAMA Cardiology*.

Test Time Augmentation with Beat-by-Beat Assessment

The SHC and Unity datasets had frame level individual annotations, while the CSMC dataset comparison was against study measurements. For PLAX measurement prediction on the CSMC dataset, at inference time, video-level augmentation was performed by aggregating predictions for each heart beat across the entire echocardiogram video. A more difficult benchmark comparison but also more reflective of opportunities for variance in clinical practice, this comparison accounts for both variation in frame selection as well as actual measurement variance. The predicted LVID measurement was used to determine frames of peak systole and peak diastole. These frames were used to generate systolic and diastolic measurements for every beat of an echocardiogram video. These measurements were compared to the human labelled measurements (Fig. 2). Variation from beat to beat in a single echocardiogram is used to evaluate the precision of the method.

Model Training and Disease Phenotyping

Patients were identified by physician curated cohorts from the Stanford Amyloid Center and CSMC Advanced Heart Disease Clinic for cardiac amyloidosis and the Stanford Center for Inherited Cardiovascular Disease and CMSC Hypertrophic Cardiomyopathy Clinic for hypertrophic cardiomyopathy. Severe aortic stenosis was identified by patients who subsequently underwent transcatheter aortic valve replacement or had a mean aortic valve gradient greater than 40mmHg. In the primary analysis, models were trained with clinical diagnoses of etiology of hypertrophy and control cases were the other etiologies of LVH. The discrimination analysis between phenotypic mimics utilized two training workflows 1) control cases of age- and sex-matched controls without selecting for LVH and 2) control cases with other types of LVH. At test time, other etiologies of LVH were introduced to see how models perform. For example, we trained a cardiac amyloid classification model with age and sex matched controls but without AS and HCM in the negative control training set, but at test time, introduced AS and HCM cases to see how frequently these cases will be confused with cardiac amyloid.

Supplemental Figures

© 2022 Duffy G et al. *JAMA Cardiology*.

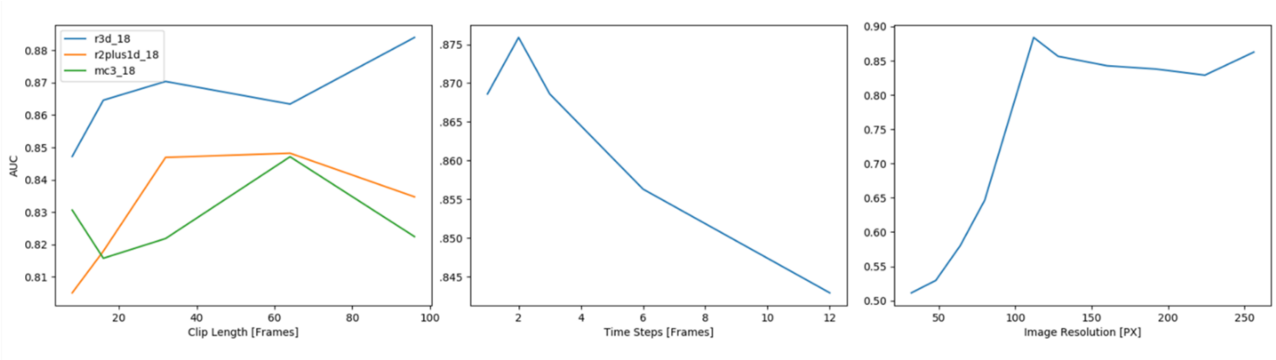

eFigure 1. Hyperparameter Search of Video-based Classification Model Hyperparameter search for model architecture (R3D, which is used by EchoNet-LVH for hypertrophy etiology classification, R2+1D, and MC3), input video clip length (8, 16, 32, 64, 96 frames) and impact on model processing time and model memory usage.

© 2022 Duffy G et al. *JAMA Cardiology*.

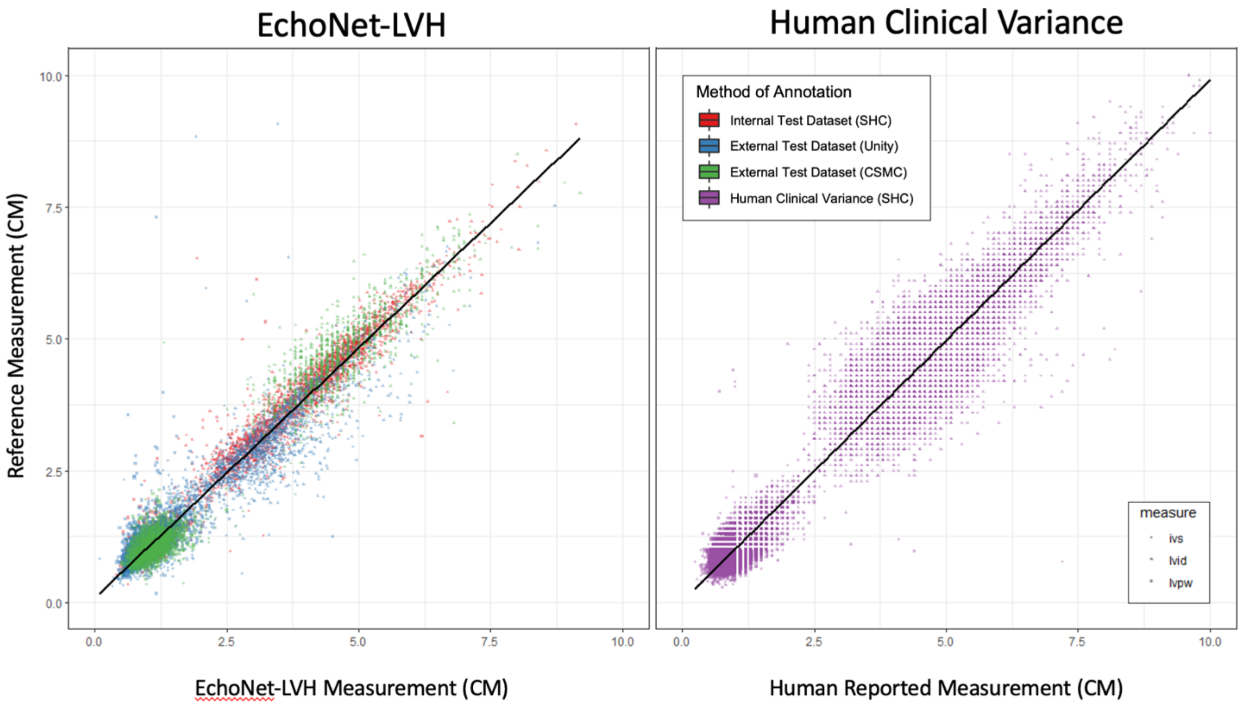

eFigure 2. Comparison of Model Performance with Human Variation A. Correlation of EchoNet-LVH measurements vs. human annotations for ventricular dimensions in datasets from three healthcare systems (n = 1,200 for SHC, n = 1,309 for CSMC, and n = 1,791 for Unity). B. Correlation of clinician reported measurements compared to prior study for studies without significant change (n = 23,874 at SHC).

© 2022 Duffy G et al. *JAMA Cardiology*.

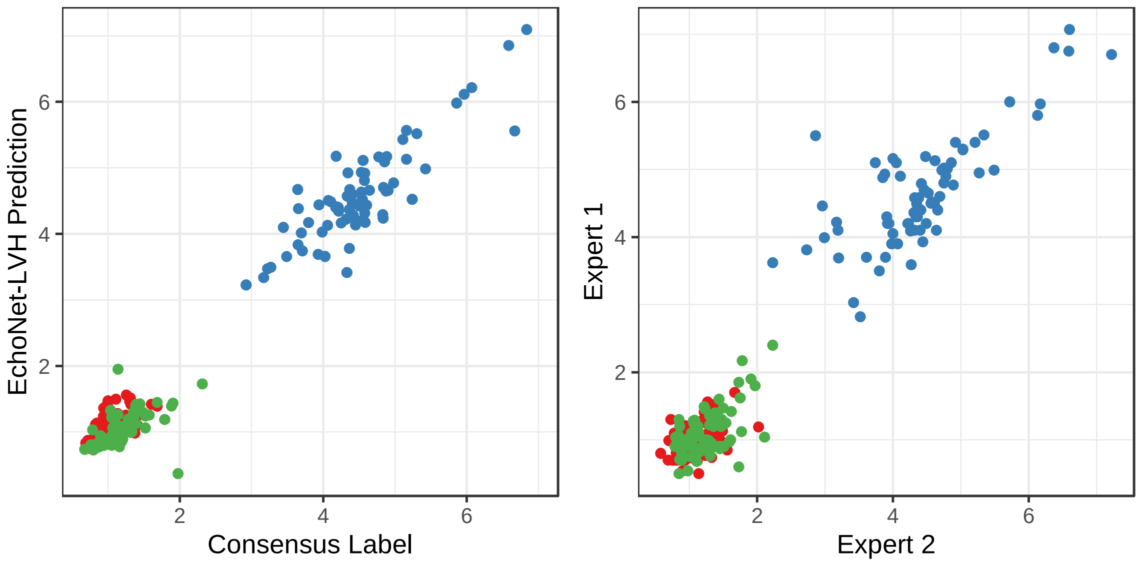

eFigure 3. Comparison of Model Performance with Prospective Consensus Annotation of Two Level III Echocardiography Certified Cardiologists.

© 2022 Duffy G et al. *JAMA Cardiology*.

Supplemental Tables

	Mean Absolute Error (mm) (95% CI)			Median Absolute Deviation (mm) (95% CI)			
Training Set for EchoNet-LVH	IVS	LVID	LVPW	IVS	LVID	LVPW	R2
Trained on SHC	2.16 (1.67-2.64)	4.51 (3.72-5.30)	2.35 (2.02-2.67)	1.34 (1.11-1.58)	2.33 (2.02-3.02)	1.62 (1.34-1.89)	0.81
Trained on Unity	1.99 (1.65-2.32)	3.08 (2.49-3.68)	2.26 (1.85-2.67)	1.26 (1.09-1.45)	1.75 (1.28-2.11)	1.61 (1.34-3.08)	0.89
Trained on SHC, Finetuned on Unity	1.74 (1.48-2.00)	2.86 (2.43-3.29)	2.26 (1.85-2.67)	1.06 (0.93-1.18)	1.69 (1.38-1.98)	1.50 (1.27-1.73)	0.92
Howard Et al.	NA	NA	NA	1.7	2.2	1.2	

eTable 1. Performance on Unity Imaging Collaborative External Test Dataset with and without fine-tuning. LVPW = Left ventricular posterior wall. LVID = Left Ventricle Internal Dimension. IVS = Intraventricular Septum.

© 2022 Duffy G et al. *JAMA Cardiology*.

	AUC			MAE (mm)		
	Amyloid	AS	HCM	LVPWd	LVIDd	IVSd
Underweight [BMI < 18.5]	0.944	0.914	0.989	0.417	1.598	0.390
Healthy [18.5 <= BMI < 25]	0.926	0.945	0.991	0.369	1.415	0.282
Overweight [25 <= BMI < 30]	0.883	0.943	0.992	0.382	1.414	0.260
Obese [BMI >= 30]	0.905	0.947	0.986	0.404	1.580	0.315

eTable 2. Performance Across Body Mass Index. On SHC test dataset. LVPWd = Left ventricular posterior wall (diastole). LVIDd = Left Ventricle Internal Dimension (diastole). IVSd = Intraventricular Septum (diastole).

© 2022 Duffy G et al. *JAMA Cardiology*.

		Trained on Matched Controls		
		Amyloid	AS	HCM
Evaluated on	Amyloid	0.95822	0.92224	0.93278
	AS	0.91805	0.97663	0.87393
	HCM	0.90786	0.89377	0.97162

		Trained with other LVH Controls		
		Amyloid	AS	HCM
Evaluated on	Amyloid	0.92283	0.24599	0.46959
	AS	0.31078	0.93272	0.35727
	HCM	0.46633	0.29025	0.98795

eTable 3. Minimzing Confusion of Alternative Etiologies of Hypertrophy When Trained on Age and Sex Matched Control Cases vs. Other Hypertrophic Control. Model trained with age and sex matched controls without selection for LVH and introduced other etiologies of LVH at test time not seen during training. Outside the diagonal, a higher AUC shows confusion of the phenotypic mimics with a high degree of inappropriate confidence.

© 2022 Duffy G et al. *JAMA Cardiology*.
